# Molecular Links and Clinical Effects of Inflammation and Metabolic Background on Ischemic Stroke: An Update Review

**DOI:** 10.3390/jcm13247515

**Published:** 2024-12-10

**Authors:** Gaetano Pacinella, Anna Maria Ciaccio, Antonino Tuttolomondo

**Affiliations:** Internal Medicine and Stroke Care Ward, Department of Promoting Health, Maternal-Infant, Excellence and Internal and Specialized Medicine (PROMISE), University of Palermo, 90127 Palermo, Italy; pacinella66@gmail.com (G.P.); amciaccio21@gmail.com (A.M.C.)

**Keywords:** ischemic stroke, diabetes mellitus, atherosclerosis, inflammation, metabolic background, oxidative stress, endothelial dysfunction

## Abstract

Stroke is a major global health concern, with 12.2 million new cases and 6.6 million deaths reported in 2019, making it the second leading cause of death and third leading cause of disability worldwide. Ischemic stroke, caused by blood vessel occlusion, accounts for 87% of stroke cases and results in neuronal death due to oxygen and nutrient deprivation. The rising global stroke burden is linked to aging populations and increased metabolic risk factors like high blood pressure, obesity, and elevated glucose levels, which promote chronic inflammation. This article explores the intricate molecular and clinical interplay between inflammation and metabolic disorders, emphasizing their role in ischemic stroke development, progression, and outcomes.

## 1. Introduction

Stroke represents a significant global health issue. The latest Global Burden of Disease surveys indicate that in 2019, there were approximately 12.2 million new cases of stroke, resulting in 143 million disability-adjusted life years lost and 6.6 million deaths worldwide [1]. This makes stroke the second leading cause of death, accounting for 12% of all deaths, and the third leading cause of disability worldwide [2].

Stroke occurs when brain tissue is locally deprived of a partial or complete blood supply due to blood vessel occlusion (ischemic stroke) or blood leakage (hemorrhagic stroke). Ischemic stroke is the most common, accounting for 87% of all stroke cases [3]. The lack of blood supply results in oxygen and nutrient deprivation, triggering a cascade of molecular events that lead to neuronal death.

Along with population aging, the global increase in stroke burden is attributed to heightened exposure to metabolic risk factors, including elevated systolic blood pressure, high body mass index, and high fasting plasma glucose [4]. All these factors contribute to the maintenance of an inflammatory status, which represents a critical mechanism underpinning stroke. In the last decades, the relationship between inflammation and metabolic background in ischemic stroke has gained much attention due to its complex role in the pathogenesis, progression, and outcomes of the disease. In this manuscript, we address from a molecular and clinical viewpoint the relevance of inflammation and dysmetabolic disorders that underlie the acute cerebrovascular event, outlining the interconnections that mutually link chronic inflammation and diseases of glucose and lipid metabolism.

## 2. Inflammation and Ischemic Stroke

Inflammation is the main contributor to atherosclerosis, which represents the primary pathological mechanism underpinning ischemic stroke [5]. Atherosclerosis is defined as plaque formation within vascular intima, characterized by subendothelial accumulation of lipoproteins, significantly oxidized low-density lipoprotein (LDL), immune cells, and extracellular matrix. The intimal thickening of arteries, followed by endothelial damage, is the “*primum movens*” of the atherosclerotic process, promoting the infiltration and accumulation of macrophages in the intima layer [6,7]. Macrophages can uptake lipids and transform them into foam cells. Indeed, lipid accumulation stimulates the expression of uptake receptors, such as scavenger receptors (SRs), on macrophages while downregulating the expression of efflux transporters, such as ATP-binding cassette A1 and G1 [8]. The continuous uptake of oxidized LDL by macrophages impairs their efferocytosis capacity and induces apoptosis. In atherosclerosis, macrophages can also undergo other forms of cell death, including pyroptosis, necroptosis, and ferroptosis [9]. The cell debris contributes to the formation of the core of the atherosclerotic plaque [10]. Vascular smooth muscle cells (VSMCs) migrate and proliferate into the intima of the arterial wall, creating a fibrous cap at the atherosclerotic plaque. The stability of the plaque depends on the thickness of the fibrous cap. In advanced lesions, the necrotic core grows while the fibrous cap diminishes, resulting in thrombosis and consequent thrombo-embolic events, including stroke.

The intimate relationship between inflammation and atherosclerosis has ancient origins and has evolved significantly over time, with essential milestones shaping our current knowledge. It dates to theories proposed by Virchow more than 160 years ago and was later developed into the “Response-to-Injury Theory” by Russell Ross [11]. Rudolf Virchow first suggested that atherosclerosis involved an inflammatory process, although this idea was not widely accepted then [12]. He regarded atherosclerosis as a chronic inflammation resulting from an active tissue reaction process, not a passive lipid deposition. However, for much of the 20th century, atherosclerosis was primarily viewed as a lipid storage disease, focusing on cholesterol accumulation within arterial walls as the critical driver. A pivotal moment came in 1973 when the pathologist Russell Ross proposed the “Response-to-Injury Theory” hypothesis, suggesting that endothelial injury is the initial event in atherosclerosis development. According to this model, endothelial injury leads to lipid accumulation, smooth muscle cell proliferation, and inflammatory response [13]. Ross argued that the immune system, specifically the recruitment of monocytes and other inflammatory cells, plays a critical role in the formation and progression of atherosclerotic plaques. By the 1990s, the idea that atherosclerosis is an inflammatory disease gained traction. In 1999, Ross, in an article published in *The New England Journal of Medicine*, described atherosclerosis as a chronic inflammatory condition [14]. He highlighted how circulating monocytes infiltrate the arterial wall and transform into macrophages, accumulating lipids and forming foam cells, the critical components of atherosclerotic plaques. Advances in molecular biology and immunology further strengthened the inflammatory theory of atherosclerosis. Researchers identified critical mediators of inflammation, such as cytokines and adhesion molecules, and highlighted the role of immune cells, such as T cells and macrophages, in plaque formation and progression. The discovery of the involvement of nucleotide-binding and oligomerization domain-like receptor family pyrin domain-containing 3 (NLRP3) inflammasome in atherogenesis further supported the importance of innate immune pathways in the pathogenesis of ischemic stroke [15]. Inflammasomes are multi-protein scaffolding complexes belonging to the innate immune system. Once activated, inflammasomes lead to caspase-1 activation and the release of interleukin-1β (IL-1β) and interleukin-18 (IL-18). The activated NLRP3 promotes endothelial dysfunction, oxidative stress, and inflammation.

Nowadays, inflammation is recognized as a central player in all stages of atherosclerosis, from the initial endothelial dysfunction to plaque formation, progression, and rupture [16]. Chronic low-grade inflammation, driven by innate and adaptive immune responses, orchestrates the complex interactions between lipids, immune cells, and the vascular wall [17]. This inflammatory process contributes to plaque development and defines the stability of plaques, with inflamed plaques being more prone to rupture, causing acute cardiovascular events such as ischemic stroke.

The endothelial lining of blood vessels functions as more than just a physical barrier between blood and surrounding tissues, acting as an organ with autocrine, paracrine, and endocrine functions. It secretes chemical mediators critical for preserving vascular homeostasis in response to various stimuli. However, several risk factors can contribute to the alteration of this protective function, leading to endothelial dysfunction, which stimulates the expression of leukocyte adhesion molecules, such as vascular cell adhesion molecule 1 (VCAM-1), intercellular adhesion molecule-1 (ICAM-1), and P-selectin on the endothelial surface. These molecules facilitate the adherence and rolling of leukocytes along the endothelium, such as T lymphocytes and monocytes, which transmigrate into the arterial intima. In the intima, monocytes differentiate into macrophages. The impaired endothelium barrier allows the entry of LDL, which undergoes an oxidative process. The oxidated LDL is internalized by the macrophages, becoming foam cells. At the same time, the inflammatory milieu induces the migration of smooth muscle cells from the media to the intima, where they accumulate lipids along with macrophages, forming early fatty streaks. The progression of this process involves the proliferation of smooth muscle cells and the deposition of collagen and other extracellular matrix components. This ongoing inflammation induces progressive remodeling of the vessel’s media and adventitia layers, forming a mature atherosclerotic plaque. The plaque comprises a lipid-rich core (atheroma) covered by a fibrous cap that separates it from the vascular lumen. Over time, the lipid core enlarges due to continuous lipid accumulation and foam cell necrosis, while the fibrous cap weakens as collagenases and matrix metalloproteinases degrade it. This process creates a vulnerable plaque that is prone to rupture. In addition, resident macrophages proliferate and secrete growth factors and proinflammatory cytokines, such as Tumor necrosis factor alpha (TNF-α), Interleukin 6 (IL-6), as well as metalloproteinase (MMP), such as MMP-1 and MMP-8, contributing to perpetuate local inflammation and favoring plaque rupture [18]. Also, neutrophils contribute to the recruitment and infiltration of monocytes in the intima by releasing reactive oxygen species (ROS) and proinflammatory cytokines, such as TNF-α, IL-1, IL-6, and IL-8.

Thus, innate immunity has a critical role in the early phases of atherosclerosis. This is followed by an antigen-specific adaptive immune response, mediated by CD4+ and CD8+ T cell activation and antibody secretion by B cells [19,20]. Specifically, T cells are among the first to be recruited into atherosclerotic plaque. Upon activation, CD4+ T cells can differentiate into various subgroups, each with distinct roles in atherosclerosis [21]. Th1 cells are pro-atherogenic by producing IFN-γ, which promotes inflammation. Th2 cells have a dual role: they may reduce inflammation and promote healing through IL-10 and IL-4 production but can also contribute to aneurysm formation. Th17 cells, through IL-17, emphasize endothelial inflammation and enhance fibrosis and plaque stability. Treg cells, known for their atheroprotective properties, secrete anti-inflammatory cytokines like TGF-β, IL-10, and IL-35, reducing inflammation and promoting plaque stability.

Chemokines play a crucial role in regulating inflammatory cell movement and promoting the development of atherosclerosis. Key pathways include the CCL2-CCR2 axis, which drives atherosclerosis progression by attracting macrophages, and the CXCL12-CXCR4 axis, which maintains endothelial integrity [22]. Recent studies also explored the role of neuroimmune cardiovascular interfaces, showing that nerve fibers tightly interact with immune cells in the arterial walls, linking the nervous, immune, and cardiovascular systems [23]. Furthermore, clonal hematopoiesis of indeterminate potential, particularly mutations in TET2 and JAK2, is emerging as a significant contributor to atherosclerosis [24]. These genetic mutations enhance inflammation and plaque instability by promoting proinflammatory responses in macrophages.

B lymphocytes play a significant role in atherosclerosis, modulating pro- and anti-inflammatory effects. They can be classified into B1 and B2 cells. B1 cells have atheroprotective effects by producing IgM antibodies against oxidation-specific epitopes and apoptotic cell antigens. On the other hand, B2 cells, which are part of the adaptive immune system, are activated by T-follicular helper cells to become plasma cells that produce IgG antibodies. B2 cells contribute to pro-atherogenic responses by secreting inflammatory cytokines and producing IgG autoantibodies. Overall, the immune response can either promote atherosclerosis or aid in healing lesions, influencing coagulation and fibrinolysis, thereby affecting the disease’s thrombotic complications.

In the 1990s, Polly Matzinger introduced the “danger theory,” shifting the view of the immune system’s primary role from distinguishing between self and non-self to recognizing and responding to danger [25]. The immune system identifies pathogen-associated molecular patterns (PAMPs) and damage-associated molecular patterns (DAMPs) from tissue damage, such as atherosclerosis. Pattern recognition receptors (PRRs) on immune cells, including macrophages and dendritic cells, detect these signals, triggering immune responses. Among PRRs, the NLRP3 inflammasome is especially crucial in atherosclerosis, linking lipid metabolism to inflammation by responding to signals including oxidized LDL and cholesterol crystals [26]. Indeed, modified lipids are regarded as DAMPs. Once activated, the NLRP3 inflammasome activates the inflammatory cytokines IL-1β and IL-18, driving atherogenesis through enhanced inflammation, recruitment of immune cells, and tissue remodeling.

Recent studies, such as the CANTOS trial, demonstrated the potential of anti-inflammatory therapies targeting interleukin-1β to reduce cardiovascular events, reinforcing the inflammatory hypothesis of atherothrombosis [27]. However, new therapies are needed to address residual inflammatory risk.

Emerging research focuses on specific immune cell populations within atherosclerotic plaques using advanced technologies like mass cytometry (CyTOF) and single-cell RNA sequencing, which reveal immune cell diversity and function. These findings could lead to novel, personalized cardiovascular immunotherapies.

Overall, a large body of literature evidence strongly supports the prominent role of inflammation in atherosclerosis. However, studying inflammation in clinical settings poses several challenges due to both the inherent complexities of inflammatory processes, i.e., peripheral vs. central inflammation, and the practical constraints of clinical research, including the timing of blood collection. Central inflammation results from activated microglia, astroglia, and T cell responses leading to local cytokine production within the central nervous system. Peripheral inflammation refers to the activation of the innate or adaptive immune system and involves systemic inflammatory biomarkers, such as proinflammatory cytokines.

The direct measurement of central inflammation is challenging because it requires invasive methods, such as cerebrospinal fluid collection or advanced imaging techniques. On the other hand, circulating biomarkers may reflect a general inflammatory state not specifically mirroring CNS pathological events, leading to potential misinterpretation. In addition, methodological and practical difficulties in studying inflammation must be mentioned. First, proinflammatory biomarkers levels can fluctuate widely according to individual factors, such as age, sex, and comorbidities, making it difficult to establish reference levels. Also, differences in analytical methods, including assay sensitivity, can contribute to inconsistent findings across studies. Another critical aspect is the timing of blood collection. Indeed, inflammation is a dynamic process, with cytokine levels changing rapidly over the time from stroke onset. Thus, the exact time of sampling is key information to avoid misinterpretation. Optimal sampling times are difficult to define and standardize across studies, as they depend on disease onset, severity, and patient-specific characteristics. Repeated sampling over time may provide a better picture of inflammation dynamics but is logistically challenging and burdensome for patients. Finally, cytokines often exhibit pleiotropic effects, playing both pro-inflammatory and anti-inflammatory effects according to the context, making it difficult to draw definitive conclusions. Table 1 summarizes the most relevant clinical studies on circulating levels of inflammatory biomarkers in patients with ischemic stroke.

## 3. Metabolic Determinants of Cerebrovascular Risk

**Figure 1:**
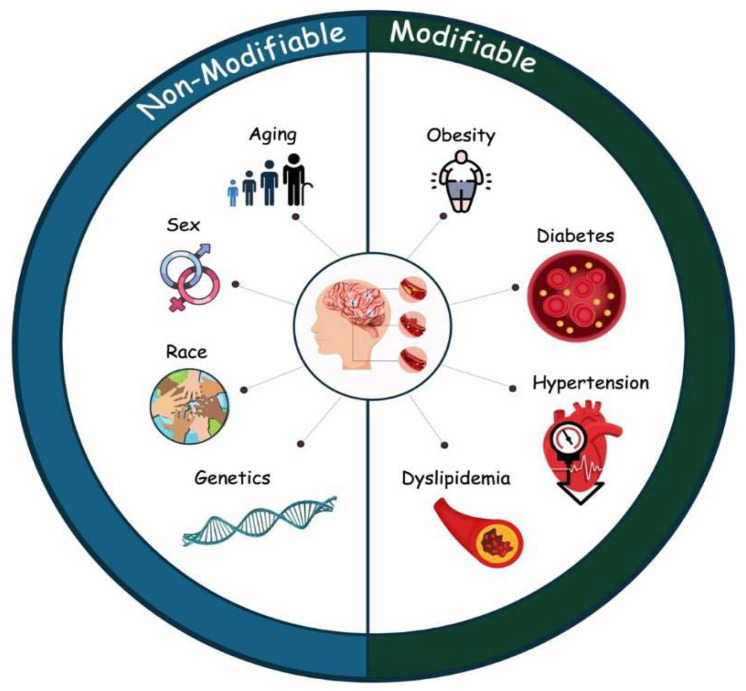
Main risk factors of cerebrovascular disease.

The endothelial dysfunction underpinning ischemic stroke is triggered by several risk factors, which can be broadly classified as modifiable and non-modifiable (Figure 1). Non-modifiable risk factors include age, sex, ethnicity, and genetics. Modifiable risk factors include metabolic determinants, such as dyslipidemia, hypertension, insulin resistance, and obesity. Addressing these metabolic determinants through lifestyle modification, such as diet, exercise, and pharmacotherapy is essential in reducing cerebrovascular risk and preventing or delaying the onset of cerebrovascular diseases.

### 3.1. Obesity

Obesity is a chronic disease regarded as an epidemic worldwide, affecting 650 million adults in 2016 and with an expected prevalence of one billion in 2030 [45]. It has been estimated that in Europe, 60% of individuals are either overweight or obese. In developed countries, nearly one-third of the population is predicted to be overweight or obese within the next few years [46].

The World Health Organization (WHO) defines being overweight and obese as having abnormal or excessive fat accumulation, globally, regionally, and in organs as ectopic lipids, which presents a health risk [47]. In clinical practice, being overweight and obese are estimated using commonly available tools, such as the body mass index (BMI). BMI is calculated by dividing the body weight in kilograms by the height in meters squared; a value over 25 kg/m^2^ indicates being overweight, and over 30 kg/m^2^ is obesity [48].

Excess food intake, low energy expenditures, and proinflammatory dietary patterns consisting of high intake contribute to the development of obesity, which is characterized by several metabolic alterations, contributing to insulin resistance, dyslipidemia, and inflammation [46]. Excess lipids and glucose initially result in adipocyte growth, proliferation, and consequent adipocyte hypertrophy, especially in visceral adipose tissue (VAT). VAT is associated with losing adipocyte metabolic homeostasis, activating resident immune cells that support tissue functions and restore homeostasis. Several innate and adaptive immune system components can be detected in visceral fat, including macrophages, dendritic cells, granulocytes, natural killer (NK) cells, and T and B cells [49]. Under physiological conditions, the resident’s immune system supports overall tissue functions. Excessive food intake and consequent calorie consumption increase the circulation of insulin. The latter is an anabolic hormone inhibiting lipolysis and promoting fat storage in adipocytes [50]. Over time, enlarged adipocytes lost the ability to preserve metabolic homeostasis of lipid storage versus lipolysis due to the lipid overload that causes endoplasmic reticulum stress, increased expression of transcription factor nuclear factor-κB (NF-κB), and proinflammatory cytokines, such as IL-6 [51,52]. Additionally, adipocytes produce leptin and other hormones with a critical role in regulating body mass via a negative feedback mechanism between adipose tissue and the hypothalamus [53]. Leptin not only reduces food intake and promotes lipolysis in visceral fat, but also stimulates leptin receptors on various immune cells, shifting the immune response towards a Th1/M1-type proinflammatory state [54].

Rapid fat tissue growth without sufficient angiogenesis in enlarged adipocytes leads to local inflammation due to reduced capillary density and oxygen diffusion, creating a hypoxic environment. Adipose tissue is highly vascularized, with each adipocyte surrounded by capillaries. When oxygen levels drop, adipocytes switch from oxidative phosphorylation to anaerobic glycolysis, altering the expression of over 1000 genes [55]. A key mediator in this process is hypoxia-inducible factor 1α (HIF-1α). Hypoxia triggers the secretion of proinflammatory mediators, such as PAI-1, CCL5, IL-6, and microRNAs. Specific macrophages support angiogenesis by producing growth factors and metalloproteinases [56]. Hypoxia does not affect visceral fat uniformly but is a regional phenomenon, often associated with increased macrophage and T cell activity, contributing to the proinflammatory environment [57].

Hypertrophic adipocytes can also promote inflammation by overexpressing major histocompatibility complex class II (MHC II) and realizing costimulatory molecules that activate CD4+ T cells. Although the specific antigens presented are unknown, studies in mice show that genetic depletion of MHC II in adipocytes prevents inflammation and insulin resistance despite weight gain [58]. Another adaptive response to metabolic stress in obese fat tissue is the conversion of white adipocytes to beige adipocytes, which have more mitochondria and burn fat for heat [59]. This process, called beiging, is promoted by IL-27 from macrophages and enkephalin peptides from type 2 innate lymphoid (ILC2) cells. Fibronectin Type III Domain Containing 4 (FNDC4), a protein released by adipocytes in obesity, has anti-inflammatory effects on macrophages [60]. Overall, the loss of metabolic homeostasis in fat tissue triggers local inflammation, with immune cells secreting proinflammatory mediators that suppress further fat storage by promoting lipolysis through different pathways, including insulin resistance and kinase activation. Additionally, enhanced lipid metabolism and thermogenesis to burn free fatty acids help reduce the adipocyte lipid load. Fat tissue cells interact with soluble mediators and extracellular vesicles, reflecting complex cellular crosstalk [61].

Beyond local inflammation, visceral obesity leads to immune cell infiltration in fat tissue primarily driven by DAMPs released by dying adipocytes. These DAMPs, along with structural damage to the extracellular matrix and the accumulation of senescent cells, trigger a complex immune response. Macrophages are critical in this process, forming crown-like structures around dead adipocytes and promoting a proinflammatory state characterized by a shift toward Th1/M1-like activity and the recruitment of various immune cells, including T and B cells, and neutrophils [62].

Senescent cells accumulate due to metabolic stress and contribute to inflammation through the senescence-associated secretory phenotype (SASP) [63,64,65]. This involves the release of proinflammatory cytokines, chemokines, and other factors that exacerbate immune cell infiltration. However, these cells can also play a role in tissue remodeling, though their prolonged presence may lead to fibrosis and impaired tissue function.

Chronic inflammation and structural damage in visceral fat can contribute to systemic metabolic disturbances, such as insulin resistance and glucose intolerance, further increasing cerebrovascular risk [29,30,31,32,33,34,35,36,37,38,39,40,41,42,43,44,45,46,47,48].

### 3.2. Diabetes Mellitus

Diabetes mellitus (DM) is a group of metabolic disorders of carbohydrate metabolism in which glucose is underutilized as an energy source and overproduced due to inappropriate gluconeogenesis and glycogenolysis, resulting in hyperglycemia [66]. It is classified conventionally into several clinical categories based on the underpinning pathophysiological mechanism. The two most prevalent forms are diabetes mellitus type I and II due to autoimmune β-cell destruction, usually leading to absolute insulin deficiency, and progressive loss of adequate β-cell insulin secretion, consequent to insulin resistance and metabolic syndrome, respectively. Impaired insulin secretion and insulin resistance also result in altered lipid metabolism with increased levels of free fatty acids. In adipose tissue, insulin suppresses lipolysis by inhibiting lipoprotein lipase (LPL). The inefficient action of insulin leads to increased LPL activity and, consequently, the release of free fatty acids. Thus, in DM, there is an intricate interaction between insulin, glucose, and FFA, which impair endothelial homeostasis through different molecular mechanisms, including oxidative stress, protein kinase C activation, and the activation of receptors for advanced products of glycosylation [67,68]. Also, the hemostatic balance is altered in diabetic patients, with a prevalence of prothrombotic status. Indeed, reduced fibrinolytic activity and the increased availability of tissue factor and factor VII have been described. Finally, chronic inflammation is another hallmark of DM.

Increased levels of NLRP3 and proinflammatory cytokines, such as IL-18 and IL-1β, have been reported in diabetic patients [69]. Hyperglycemia also promotes neutrophil extracellular traps (NETs) activation and release (NETosis). NETosis represents a unique form of cell death first described in neutrophils and then in other granulocytes, characterized by the expulsion of chromatin during cell death. Unlike apoptosis (programmed cell death) or necrosis (uncontrolled cell death), NETosis involves the release of NETs, which consist of decondensed chromatin (DNA and histones) and antimicrobial proteins trapping and neutralizing pathogens, such as bacteria, fungi, and viruses. While NETosis is a vital defense mechanism, the DAMPs released during NETosis act as potent inducers of inflammation. Studies in animal models revealed that NETosis can also directly promote atherosclerosis [70]. NET-derived extracellular DNA and protein components have cytotoxic and proinflammatory functions, providing a causal link between hyperglycemia, inflammation, and stroke [71].

Overall, diabetes mellitus is an important and independent contributor to cerebrovascular risk.

### 3.3. Hypertension

Hypertension, defined as a systolic blood pressure value of ≥130 mm Hg and/or diastolic blood pressure > 80 mm Hg, is universally recognized as the most important modifiable risk factor for stroke [72,73]. The INTERSTROKE study, a large, standardized case–control study including 3000 stroke patients and 3000 controls from 22 countries worldwide, showed that hypertension accounts for more than 80% of the global risk of strokes [74].

It has been estimated that 26% of the world’s population has hypertension, making it a tremendous public health burden. At any age, increased mean blood pressure is associated with an elevated risk of stroke, with an age-dependent effect [75]. Indeed, hypertension prevalence increases with age, from 46% at 20–44 years to >78% in over 65 years [76].

The pathophysiological mechanisms underpinning the relationship between hypertension and increased cerebrovascular are complex and involve intricate interactions between vascular, neural, and systemic processes. It is well established that hypertension impacts cerebral blood vessels’ structure and function, leading to endothelial damage and thickening of the arterial walls, respectively [77]. Endothelial function is regulated by various mediators, including nitric oxide (NO) and prostaglandins, as well as mechanical stimuli like vascular shear stress. Laminar flow promotes vasodilation and vascular protection by activating mechanosensitive pathways, increasing NO production, and suppressing inflammation. It enhances endothelial integrity through glycocalyx mechanosensing and other signaling cascades involving integrins and cytoskeletal components. In contrast, oscillatory flow reduces NO expression and promotes proinflammatory processes, such as leukocyte infiltration and reactive oxygen species (ROS) production, leading to vasoconstriction, increased blood pressure, and atherosclerosis [78,79]. This flow pattern activates several transcription factors and pathways contributing to endothelial dysfunction, vascular remodeling, and cardiovascular complications.

Much evidence shows that ROS play a significant role in endothelial dysfunction, especially hypertension [80]. Under hypertensive conditions, the primary source of ROS in the vascular system is NADPH oxidase, which is upregulated in response to factors such as altered shear stress, renin-angiotensin system (RAS) activation, and endothelin [81]. ROS act as signaling molecules for vasoactive agents like angiotensin II and endothelin-1, influencing cellular processes through redox-sensitive pathways. This leads to changes in calcium homeostasis, promoting vasoconstriction, cell growth, inflammation, and hypertension-related organ damage.

ROS also activate several signaling pathways involved in inflammation and vascular remodeling, such as NF-κB, MAPKs, and STAT, contributing to oxidative stress [82,83]. This results in aberrant signaling and post-translational modifications, including the oxidation of proteins, which drives cell and tissue damage. In addition, ROS can inhibit protective enzymes like SIRT1 and lower NO bioavailability, further exacerbating endothelial dysfunction and promoting hypertension [84].

**Figure 2:**
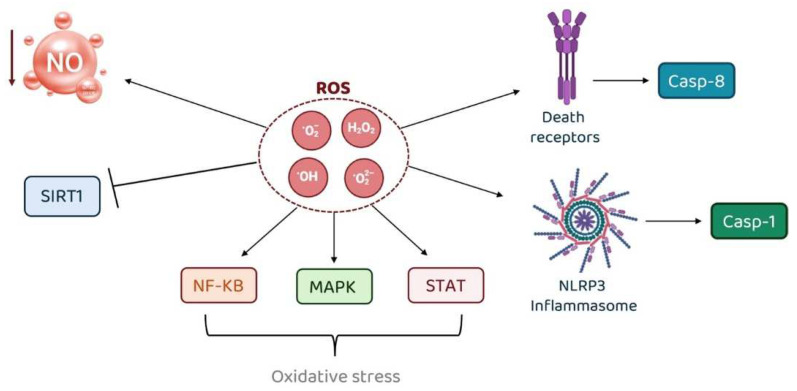
Molecular pathway linking ROS to inflammation.

An intricate interplay between ROS and caspase has also been described. Caspases, a family of proteases, mediate the downstream effects of ROS by activating inflammatory pathways and orchestrating cell death processes, such as pyroptosis and apoptosis. Specifically, ROS indirectly activate caspase-1, through the stimulation of NLRP3 inflammasome, and caspase-8, through the activation of death receptors, such as Fas or TNFR1. Caspase-1 induces pyroptosis, a pro-inflammatory form of programmed cell death that releases cytokines and DAMPs, while caspase-8 initiates apoptotic pathways and modulates inflammation by directly interacting with NF-κB and other signaling cascades or indirectly by releasing intracellular DAMPs during apoptotic cell death. In turn, caspase-mediated cell death causes mitochondrial dysfunction, leading to the release of additional ROS, creating a positive feedback loop, sustaining inflammation. ROS damage cellular components, resulting in the release of DAMPs that activate caspases. Caspase activation amplifies inflammatory signaling, perpetuating a cycle of oxidative stress and inflammation. ROS and caspases are key mediators of inflammation, with ROS triggering caspase activation and caspases propagating inflammatory responses. Their interplay creates a vicious circle that underpins many inflammatory diseases, highlighting them as critical targets for therapeutic intervention. Figure 2 describes the pathways linking ROS to inflammation.

A hallmark of hypertension is an increased media-to-lumen ratio or wall-to-lumen ratio in microvessels due to a narrowing of the internal lumen and an increase of tunica media or total wall thickness, resulting in arterial stiffness, defined as a reduced elasticity of the arteries [85,86,87]. Stiffer arteries can increase shear stress on the arterial wall, accelerating the process of atherosclerosis.

Finally, hypertension can impair the integrity of the blood–brain barrier (BBB), increasing permeability and allowing toxins, immune cells, and other molecules to infiltrate the brain. This can trigger inflammation and oxidative stress, further damaging brain cells and contributing to cerebrovascular risk [88].

### 3.4. Dyslipidemia

Dyslipidemia is a significant risk factor for cardiovascular disease [89]. It is characterized by increased levels of lipoproteins and related lipids due to altered lipid metabolism or function. According to the etiology, dyslipidemia can be classified as primary due to genetic alterations in gene codifying for molecules involved in lipid metabolism or secondary to other clinical conditions, such as diabetes or hypothyroidism. Dyslipidemia represents a global public health problem, with a global prevalence ranging from 20% to 80%, depending on the definition and criteria used [90].

The relationship between dyslipidemia and cardiovascular risk is multifaceted, involving a complex interplay of biochemical, cellular, and physiological processes contributing to atherosclerosis development.

First, increased levels of LDL, as in familiar hypercholesterolemia, the most common form of primary dyslipidemia, promote their migration and deposition in the intima of atheroprone sites, triggering the cascade of events that result in the formation of atherosclerotic plaque. LDLs trapped in the arterial intima undergo chemical modification, especially oxidation. Literature evidence reveals that LDL promotes oxidative stress within endothelial cells [91]. Specifically, LDL contributes to ROS production through both enzymatic reactions, involving lipoxygenases, myeloperoxidase, and cyclooxygenases, and non-enzymatic processes mediated by NADPH/NADH oxidase or uncoupled eNOS [92].

The oxidation of LDL results in the formation of oxidation-specific epitopes (OSEs) on the oxidized LDL. OSEs are recognized by various receptors such as scavenger receptors, toll-like receptors, complement system mediators, and natural IgM antibodies. Studies indicate that inhibiting lipoxygenases genetically can reduce LDL oxidation and slow atherosclerosis in mice [93]. Similarly, natural IgM antibodies specific to OSEs can block LDL uptake by macrophages, preventing the formation of foam cells, a critical step in the initiation of atherosclerosis [94].

Moreover, endothelial cells and macrophages actively take up oxidized LDL, contributing to its proatherogenic effects.

Overall, oxidative LDL contributes to the development and progression of atherosclerosis through pleiotropic effects, by stimulating the expression of adherence molecules on the endothelium, activating macrophages and T cells, and inactivating the eNOS [95].

On the contrary, low high-density lipoprotein (HDL) levels independently predict cerebrovascular risk [96]. Beyond the inverse cholesterol transportation, HDL has several beneficial effects on endothelial function, including the activation of eNOS, the reduction of ROS, adhesion molecules, and chemokines levels, and the attenuation of inflammation [95]. Thus, reduced HDL levels are associated with losing all these beneficial effects and an increased predisposition to developing cerebrovascular diseases [97].

## 4. “Metabolic Players” in the Inflammatory Cascade of Ischemic Cerebrovascular Accident

Although several underlying molecular mechanisms can be identified, the metabolic cornerstones of acute cerebral vasculopathy belong to two major diseases that, from an epidemiological point of view, represent the real health emergency of the 21st century: type 2 diabetes mellitus (T2D) and atherosclerosis.

As far as T2D is concerned, apart from the definition and description of the main pathophysiological processes characterizing it already described above, it should be pointed out that the careful study of the molecular mechanisms leading to the destruction of pancreatic β cells and the complete dysregulation of glucose metabolism may enable the link between this dysmetabolic disorder and ischemic stroke to be fully understood: it appears that in a condition of nutrient excess (a condition that occurs in patients with obesity and T2D), the surplus of glucose and lipids increases oxidative stress, which stimulates a state of chronic inflammation that promotes cellular damage within the pancreas [98].

The storage of glycidic and lipid substrates leads to an alteration of the endoplasmic reticulum by triggering an apoptotic molecular pathway of the unfolded protein response pathway (UPR): cellular homeostasis is thus disrupted through inhibition of the sarco/endoplasmic reticulum Ca2+ ATPase (SERCA) that regulates intracellular calcium and activation of receptors for 1-4-5 inositol triphosphate (IP3) [99].

Among other things, excess glucose causes an increase in the production of proinsulin and pancreatic islet amyloid polypeptides (IAAPs), which, as they accumulate, accelerate the oxidative stress we have already mentioned. The combination of these aberrations impairs the normal mobilization of calcium from the endoplasmic reticulum to the cytoplasm. It corresponds to activating an apoptotic cell death signal, with release into the systemic circulation of a potent inflammatory cytokine such as interleukin-1 beta (IL-1β). What has been described so far removes the feedback that insulin and glucagon exert on glucose metabolism, causing an imbalance in favour of hyperglycemia that further catalyzes the abnormalities described as a vicious cycle [100].

Suppose this decline begins in the pancreatic islets and is the germ of the damage that T2D produces at the systemic level. In that case, it must be considered that the chain of molecular alterations continues and does not stop: once hyperglycemia sets in, the complications of this condition impact the target organs; at the level of the great arteries, for example, hyperglycemia (in concert with other risk factors such as cigarette smoking and dyslipidemia) causes platelet dysfunction and an uncontrolled conversion of fibrinogen to fibrin, which increases the risk of thrombotic events and the evolution of any pre-existing atheromasic plaques, a situation of great potential effect on the cerebral vascular district in terms of ischemic events. At the microvascular level, on the other hand, the damage produced by oxidative stress and hyperglycemia affects the vascular endothelium (predominantly in organs such as eyes and kidneys): not surprisingly, in the diabetic patient, diabetic retinopathy is the most frequent cause of visual impairment and blindness, just as chronic kidney disease and its evolution to terminal stages with the need for hemodialytic replacement treatment are among the most impactful issues in terms of public health [101].

However, the aspect of understanding neuropathic and vascular tissue complications goes beyond what we have said so far: nerve and endothelial cells do not have receptors for insulin, which physiologically determines the internalization of glucose into cells and regulates its concentration, so glucose levels in these cells are the same, by osmosis, as in plasma; it follows that in the case of hyperglycemia, these cells will have excess glucose in their cytoplasm, and will attempt to dispose of it through metabolic pathways additional to anaerobic glycolysis and the Krebs cycle (polyol pathway, nonenzymatic glycation of substrates, for example), causing the failure of cytoplasmic elements and cell membranes, with deterioration of the cell as a result, ultimately [102].

The effects of pancreatic damage on glucose metabolism are only some of the effects that occur over time: insulin is also a regulator of lipid metabolism since it acts at the adipocyte level and on free fatty acid levels by promoting lipogenesis; when damage to beta cells is established and insulin deficiency is consolidated in patients with long-term diabetes mellitus, the excess of free fatty acids and the perpetuation of the oxidative stress this entails accelerate vascular deterioration and thus contribute to the increased risk of ischemic vascular events [68].

If the main metabolic determinant of cardiovascular risk is diabetes mellitus, the other side of the coin is atherosclerosis, which can trigger inflammatory molecular processes that result in vasculopathy. We have already mentioned the pathogenesis of atherosclerosis and the role of hyperlipidemia and endothelial damage in the development of intimal plaque. However, it is crucial, in order to fully understand the role of atherosclerosis as a key player in vasculopathy, cerebral and otherwise, to dwell on the “inflammatory” consequences of the atherosclerotic burden: in the atherosclerotic plaque, there is a recruitment of leucocytes from the bloodstream produced by the presence of oxidized LDL and the monocyte chemotactic protein type 1 (MCP-1); the recruited monocytes differentiate into macrophages, which introduce the oxidized lipoproteins through “scavenger” receptors (SR-A and CD36) and this process continues until the death of the cell, since these receptors, unlike the receptors for unoxidized lipoproteins, do not have a feedback regulation mechanism and do not block the storage of lipid substrates. The death of these lipid matrix-loaded macrophages by necrosis and apoptosis eventually expands the lipid core of the plaque and makes it more extensive and unstable [103].

A potentially interesting issue is that macrophages, in addition to expanding the plaque, make it unstable and harmful as they produce protein-degrading enzymes (metalloproteinases) able to damage the extracellular matrix, thus leading to exposure of the lipid core, platelet activation, and thrombotic events with fibrin activation that are the “primum movens” of ischemic thrombo-embolic-based vascular accidents. Therefore, since macrophages constitute a valiant soldier of the immune system in protecting organisms from infectious agents, they become the worst enemy of arterial vessels in this degenerative process [104].

This process involves other cells because the tissue damage that occurs draws in and activates vascular smooth muscle cells (VSMCs), which can change their phenotype depending on the environmental stresses to which they are subjected and, on the one hand, play a protective role as they work to repair the damage to the artery through a fibroproliferative mechanism. However, on the other hand, they can play a destructive and destabilizing role in the plaque for a variety of reasons; meanwhile, if the atherogenic drive continues, the fibroproliferative repair process may continue unconditionally and reduce the vessel diameter at that point by reducing blood flow, and then with the expression of macrophage-like and foam cell-like phenotypes, they may also have a destabilizing role [105].

**Figure 3:**
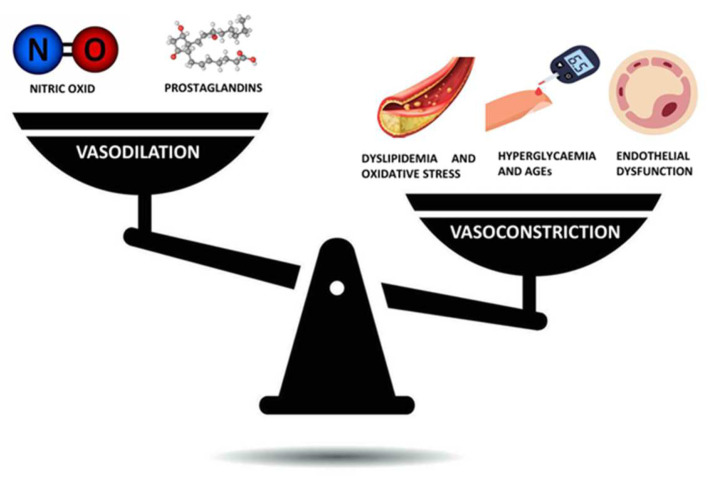
Alteration of the balance between vasoconstriction and vasodilation leads to vascular pathology.

The altered metabolic background that characterizes T2D and atherosclerosis, therefore, constitutes the most critical pathophysiologic substrate and it is marked by a series of effects that arise from those molecular “aberrations” that have a disastrous impact on the health of the vascular tree: endothelial dysfunction, smooth muscle cell alterations, platelet abnormalities, and coagulation alterations. Regarding endothelial dysfunction, it plays a significant role in the pathogenesis of cardio- and cerebrovascular diseases: the endothelium is the inner layer of vessel and in addition to its function as a barrier, it has an “endocrine” role by producing biologically active compounds that can modify the balance between vasoconstriction and vasodilation (nitric oxide, prostaglandins, endothelin-1, other reactive oxygen species) (Figure 3).

We have already described the mechanism of nitric oxide (NO) production as a vasodilator and inhibitor of platelet activation, and, thus, as a promoter of vascular health; it happens, however, that in diabetic patients, hyperglycemia inhibits the activity of the enzyme endothelial nitric oxide synthetase (eNOS), causing a reduction in the bioavailability of NO, and concomitantly, due to increased oxidative stress, there is an upregulation of other reactive oxygen species including superoxide anion (O^2−^) that have a negative and harmful role [106].

Superoxide anion, in turn, counteracts NO production through the production of the toxic ion peroxynitrite, which can uncouple the enzyme eNOS from its cofactor tetrahydrobiopterin and thus unbalance vascular homeostasis toward vasoconstriction (Figure 3) [107].

Reduced insulin activity in adipose tissue also implements this process: the excess of saturated fatty acids results in activation of the protein kinase C pathway and subsequent inhibition of phosphatidyl-inositol-3 (PI-3) phosphate kinase, which leads to an increase in reactive oxygen species at the expense of nitric oxide [108].

The imbalance towards vasoconstriction in diabetic patients depends not only on the lack of production of vasodilator mediators but also the increased production of vasoconstrictor ones: endothelin-1, for example, promotes smooth muscle cell hypertrophy through activation of the RAAS system (renin–angiotensin–aldosterone system), with consequent retention of water and salt. Moreover, serum levels of endothelin-1 in these patients are also higher in response to the insulin action of modulating gene expression and receptor synthesis, triggering a vicious circle that amplifies vascular constriction [68].

Other molecular players in the inflammatory cascade are undoubtedly the advanced glycosylation end-products (AGEs): excess glucose in the blood of diabetic patients leads to an increase in non-enzymatic glycosylation of extracellular proteins, which, by accumulating, causes worsening of endothelial damage, as endothelial cells increase the adhesion molecules on their surface and emphasize the chemotaxis of monocytes/macrophages, which converge on the site where the atherosclerotic plaque is forming and increase inflammation by promoting the release of inflammatory cytokines [109].

Atherogenesis and the vascular ischemic damage that follows are, therefore, the result of a coincidence of molecular pathways that culminate in the maximal promotion and exacerbation of inflammation that represents the “wrecker of arterial vessels”: hyperglycemia, oxidative stress, and AGEs at the nuclear level trigger the increased transcription of the nuclear factor kappa-light-chain-enhancer of activated B cells (NF-kB); this results in the increased production of inflammatory cytokines such as interleukin 1 (IL-1), increased expression of adhesion molecules, and maximal escalation of atherogenesis that results in the acute cerebrovascular accident.

However, the risk of plaque rupture in the atherogenesis progression also depends on other factors, among which platelet dysfunction plays a significant role. Like endothelial cells, platelets do not have insulin receptors, so the intraplatelet glucose concentration is the same as in plasma, with similar consequent degenerations already described for endothelial cells (protein C activation, reduced NO production, increased ROS), from which follows the impairment of platelet function by the dysregulation of calcium transport and thromboxane synthesis. Among the platelet aberrations in diabetic patients, there is an increase in glycoprotein Ib and glycoprotein IIb/IIIa surface expression, which is responsible for interaction with fibrin and thus enhances prothrombotic risk [110].

In the evolution of the thrombotic mechanism that follows atheromasic plaque breakdown, the platelet dysfunction just described represents the first stage: some studies have shown that these patients have reduced fibrinolytic activity due to an increase in tissue factor plasminogen type 1 inhibitor both at sites of atherogenesis and at arterial vessels not affected by the atheromasic process [111].

Thus, in addition to the imbalance toward vasoconstriction, the vascular risk profile in patients with diabetes and atherosclerosis also depends on the imbalance toward hypercoagulability through the loss of coagulative homeostasis (which certainly depends in part on hyperglycemia but could also depend on the increased release of proinsulin degradation products in the mechanism of conversion to insulin) [112].

There are many mechanisms involved in the pathogenesis of stroke, regardless of subtype, and recently some trials have underlined the duality of “post-stroke” inflammation owing to the fact that inflammation contributes both negatively and positively to neurological outcomes [113,114,115]. Brain ischemia triggers inflammation as a response against necrotic cells followed by the generation of reactive oxygen species (ROS), although many other factors have yet to be identified. Once activated, these initiators of inflammation lead to activation of microglia, the brain’s resident immune cells. The microglia then generate more pro-inflammatory cytokines, which in turn leads to adhesion molecule induction in the cerebral vasculature [116,117,118,119]. These inflammatory humoral recruitments have been documented to occur within the first 24 h of the stroke onset. Cytokine upregulation leads to the chemotaxis of circulating immune cells into ischemic brain, especially around the ischemic “penumbra”.

Adhesion molecules facilitate leukocyte adhesion to the vascular endothelium, resulting in microvascular occlusion and leukocyte infiltration into the ischemic brain parenchyma [120,121].

Although the importance of metabolic status in patient outcome is probably noteworthy, the metabolic response to stroke and the role of metabolic variables regulating immunoinflammatory activation after an ischemic stroke has undergone little preclinical or clinical investigation. Therefore, the study of global metabolism to understand metabolic differences associated with stroke and the influence of obesity on outcome is an important research area. Metabolites are important biochemicals actors because they act as precursors for the synthesis of other biochemical components(e.g., proteins, RNA, DNA) and cellular structures (e.g., cell walls).

Furthermore, ischemic stroke is characterized by the presence of reactive microglia. Once activated, the microglia enter a chronic tolerant state as a result of widespread energy metabolism abnormalities, which reduces immunological responses, including cytokine release and phagocytosis. Metabolically impaired microglia cells were identified in mice through genome-wide DNA sequencing following chronic chemokine-like factor 1 (CKLF1) administration, which was accompanied by a reduction in the inflammatory response.

Metabolic conditions such as diabetes and metabolic syndrome could reasonably be considered potential triggers capable of influencing the post-ischemic metabolic shift in the microglia population [122].

In a study of our own group (unpublished data), we observed a relationship between serum levels of glucose and total and LDL cholesterol in patients with more severe ischemic stroke. In addition, we found a significant association between all the immunoinflammatory variables and LDL-cholesterol and serum glucose levels. Patients with elevated LDL-cholesterol levels showed significantly higher levels of TNF-α, IL-1, and IL-6, along with lower IL-10 levels, compared to those with normal LDL-cholesterol levels. Similarly, patients with elevated blood glucose levels exhibited significantly reduced IL-10 levels and increased TNF-α, IL-1, and IL-6 levels compared to individuals with normal blood glucose levels. These findings appear to be both original and novel.

Proinflammatory cytokines such as TNF-α, IL-6, and IL-1 have been shown to disrupt insulin and lipid signaling pathways, thereby affecting insulin sensitivity and lipid metabolism. Notably, numerous studies have demonstrated that TNF-α contributes to insulin resistance through various mechanisms. These include downregulating key genes essential for insulin’s biological functions, such as GLUT-4, promoting an increase in free fatty acid levels by stimulating lipolysis, and negatively regulating peroxisome proliferator-activated receptor-γ (PPAR-γ).

It has been shown that in pancreatic β cells, IL-1 can activate the Jun amino-terminal kinase (JNK) pathway, which mediates the suppression of the transcription of the insulin gene. In addition, IL-1 causes a reduction in the expression of insulin receptor substrate 1 (IRS-1), the inhibition of the translocation of the glucose transporter GLUT-4 to the plasma membrane, and the reduction of the absorption of stimulated glucose insulin and lipogenesis. In mouse models, IL-1 has been found to play a dual role. On one hand, it promotes hepatic steatosis by stimulating triglyceride production, cholesterol accumulation, and lipid droplet formation. On the other hand, it regulates hepatic insulin resistance and fibrosis. Conversely, IL-1 inhibition has been found to attenuate steatosis and liver damage, improve atherosclerosis, and reduce circulating glucose levels. IL-6 appears to regulate insulin sensitivity through multiple mechanisms. It has been shown to exert long-term inhibitory effects on the transcription of IRS-1, GLUT-4, and PPAR-γ genes, as well as on insulin-stimulated tyrosine phosphorylation and glucose transport. These actions collectively impair insulin signaling and reduce its effectiveness.

## 5. Impact of Metabolic Background on the Prognosis of Ischemic Stroke

The metabolic milieu of the ischemic stroke patient is an indispensable factor not only with regard to the onset of vascular damage but certainly also in terms of survival from the time of the cerebrovascular event [123,124]. Literature data, in fact, report that patients with diabetes mellitus have a higher mortality rate after ischemic stroke than non-diabetic patients: some works report a mortality rate at least three times higher within the first six months of the event in the former than in the latter [125]; other trials show that in diabetic patients with poor glycemic control, the prognosis is unfavorable and the mortality impact after ischemic stroke is already higher within the first thirty days after the event [126].

The reasons concerning the relationship between poor glycemic control and increased mortality can be found in several factors: the first and perhaps most obvious one concerns poor compliance with medical therapy, since patients with bad glycemic balance and thus higher glycated hemoglobin (HbA1C) values almost always have an unhealthy lifestyle, which in itself constitutes a risk for high mortality after an ischemic event. Secondly, glucose transport across the blood–brain barrier is impaired during the acute phases of the ischemic event, resulting in hyperglycemia and a series of metabolic, dysautonomic and hormonal alterations that may worsen the ischemic event during the acute phase [127]. An additional element is surely atherosclerosis of the cerebral vessels: the atherosclerotic burden that is inextricably linked to the impaired metabolic background causes chronic blood flow alterations and thus a reduced power to contain the ischemic core, which consequently expands with reduced chance of survival and worse residual disability, affecting the brain district and also involving other organs and systems.

In addition to glycemic control, the duration of diabetes also has a great impact on the prognosis of the patient with ischemic stroke: long-standing disease inexorably affects the prognosis of individuals who have experienced an acute cerebrovascular accident as opposed to diabetes per se, and this is mainly dependent on the establishment of disease complications as it progresses [128].

From a molecular point of view, the duration of diabetes is so relevant because it worsens endothelial dysfunction through a reduction in nitric oxide bioavailability and an imbalance toward vasoconstriction that is not observed with the same severity in patients with short-term diabetes, and this promotes the recurrence of ischemic events and thus mortality [129].

As the duration of diabetes increases, excess reactive oxygen species that exacerbate oxidative stress, activation of the protein kinase C pathway, and advanced glycosylation end-products permanently impair the autoregulation of the cerebral blood circulation, with the progression of atherosclerotic damage and worsening of atheromasic plaques: the recurrence of ischemic events after the first stroke results in a spiral decline that impacts the survival of the patient in no small proportion [130].

Diabetes, however, is a systemic disease and, as such, also produces damage to target organs like the kidney: microalbuminuria in patients with long-standing diabetes is present to a greater extent and also tends to increase, and since several trials have shown it to be an independent risk factor for ischemic stroke in diabetic patients, we can conclude that it represents an essential element to consider in terms of recurrence of the ischemic event and thus survival [131].

In attempting to scan the prognosis of the ischemic stroke patient, in addition to the survival and recurrence data of ischemic events we have mentioned, we cannot ignore the cognitive impairment these patients often experience: cognitive decline occurs in 25–30% of stroke survivors, and may occur in a short time or at a longer distance resulting in vascular dementia [132].

In recent years, among other things, researchers have found that disease duration and glycemic control are important factors in affecting the prognosis of ischemic stroke patients with T2D, but glucose variability also has a considerable impact: excessive variability in plasma glucose concentration and fluctuations between very wide thresholds appears to be associated with the development of post-stroke cognitive impairment (PSCI) in patients experiencing this condition; it also appears that some regions of the brain are more vulnerable to this phenomenon such as the frontal region, memory centers and those overseeing executive functions. Increased plasma concentrations of receptors for advanced glycosylation end-products (RAGE), particularly soluble ones, appear to be a trigger for the development of vascular dementia [133].

It is not surprising, therefore, that subjects with ischemic stroke and diabetes have worse cognitive performance than non-diabetic patients only 3-6 months after the cerebrovascular event. The mechanism of this may depend on the proinflammatory microenvironment that diabetes mellitus, in concert with atherosclerosis, promotes and on the fact that the inflammatory trigger may accelerate the progression of neurodegenerative diseases that result in cognitive decline [134].

Patients with ischemic stroke comorbid with diabetes have higher in-hospital mortality and are more often discharged to nursing homes than non-diabetics. In addition to functional problems and physical disability, these patients more often experience depression, which, therefore, should be considered as impacting their prognosis [135].

Dementia and depression mutually influence each other; if the patient had dementia before the ischemic event (pre-stroke dementia), the cerebrovascular event worsens the extent of dementia. It promotes further cognitive impairment, assuming, in this case, that the patient may have had an underlying neurodegenerative disorder before the stroke [136].

## 6. Conclusions

The current scientific literature, as the state of the art, provides us with a clear picture: metabolic disorders, notably atherosclerosis and diabetes mellitus, because of their epidemiological impact and the multifaceted alterations they cause, play a fundamental role in ischemic stroke: they are the “primum movens” of cerebral vasculopathy since they cause those molecular dysregulations that promote endothelial dysfunction, oxidative stress, vasoconstriction, and coagulation disorders, and thus represent the causative elements of the ischemic event. However, just as clearly, they affect the prognosis of the patient who experiences an acute cerebrovascular accident: the data on survival, cognitive impairment, depression, and vascular dementia that these patients have to face suggest that the impact of these diseases does not end with the ischemic stroke (which albeit seems to be the catastrophic conclusion of the sequence of the aberrations such as those that we have described above), but continues inexorably to the point of representing a disadvantage in terms of survival and a risk factor for further comorbidities. The efforts of modern medicine, therefore, should be aimed at prevention: preventing T2D and atherosclerosis not only means reducing the risk of ischemic stroke, but also means improving the survival of those who experience acute cerebrovascular events, reducing the incidence of depression and cognitive impairment, and lowering the likelihood of developing vascular dementia.

The metabolic background thus constitutes the beginning and the end of our reasoning; it is the framework within which these pathologies unfold and on which their severity depends. Tailored patient therapy, which in the 21st century has gained increasing importance, aims to counteract the effects of these diseases by opposing the activation of specific pathways involved, and scientific research is expanding in the same direction to attack more and more precisely the molecular targets responsible.

## Figures and Tables

**Table 1:** Most relevant clinical studies on circulating levels of inflammatory biomarkers in patients with ischemic stroke.

Authors and Year Pubbl.	Study Design	Study Population	Main Findings	Conclusions
NLRP3				
Wang et al., 2022 [28]	Observational retrospective case–control	60 patients with AIS and 20 healthy subjects	The levels of NLRP3 were higher in patients than controls. In patients, NLRP3 was associated with poor prognosis	NLRP3 could provide prognostic information in patients with AIS.
Sun et al., 2022 [29]	Observational retrospective case–control	45 patients with NVAF and 60 patients with NVAF and AIS	Patients with AIS had high levels of NLRP3. NLRP3 in AIS patients was positively correlated with CHA2DS2-VASc score. ROC showed that combined detection of NLRP3, IL-6, CRP, and CHA2DS2-VASc in predicting NVAF complicated with IS exhibited an area under the curve of more than 0.9.	NLRP3 is a predictor of NVAF complicated with IS.
Wang et al., 2021 [30]	Observational prospective case–control	200 patients with AIS, of whom 26 patients developed MBE	Patients with MBE had higher levels of NLRP3. NLRP3 level was positively correlated with NIHSS on admission	NLRP3 is a predictor of complications of AIS.
TNF-alpha				
Duan et al., 2022 [31]	Meta-analysis	9120 IS patients and 9249 healthy controls	The level of TNF-α cytokine was elevated in IS patients compared with controls.	Increased levels of TNF-α are involved in the etiology of IS.
Loga-Andrijić et al., 2021 [32]	Observational prospective case–control	100 AIS patient and 30 controls with discogenic lumbosacral radiculopathy	AIS with cognitive impairment had significantly higher levels of TNF-α than those without cognitive impairment	TNF-alpha is associated with worse prognosis in AIS patients.
Wu et al., 2019 [33]	Meta-analysis	9 case–control studies	Elevated circulating level of TNF-α in IS patients	TNF-alpha could be involved in IS pathogenesis.
CCL2-CCR2				
Georgakis et al., 2019 [34]	Meta-analysis	17,180 individuals’ stroke-free at baseline, followed for a mean interval of 16.3 years. A total of 1435 incident stroke cases were diagnosed during follow-up, which were classified as ischemic in 1233 cases and as hemorrhagic in 205 cases.	Higher levels at baseline were associated with a higher long-term risk of stroke after accounting for age, sex, race, and vascular risk factors. Considering stroke subtypes, the levels were associated with the risk of ischemic, but not with hemorrhagic stroke	Increased levels of CCL2 are associated with increased long-term risk of stroke, suggesting that MCP-1- signaling might represent a therapeutic target to lower stroke risk.
CXCL12/CXCR4				
Cheng et al., 2017 [35]	Observational prospective	304 patients with AIS	Patients with an unfavorable outcome and non-survivors had significantly increased CXCL12 levels on admission. CXCL12 was an independent predictor of functional outcome and mortality with an AUC of 0.80 and 0.87, respectively.	Circulating CXCL12 serum levels at baseline are a useful biomarker to predict functional outcome and mortality 6 months after AIS.
Duan et al., 2012 [36]	Observational prospective	288 patients with AIS	CXCL12 levels > 3.5 ng/mL levels predicted AIS with an AUC 0.907. additionally, CXCL12 levels ≥ 7.6 ng/mL were associated with an increased risk of unfavorable outcome.	Increased levels of CXCL12 at admission are an independent diagnostic and prognostic biomarker in patients with AIS.
IL-6				
Zhang et al., 2022 [37]	Retrospective cohort study	116 patients with arterial atherosclerotic ischemic stroke divided into poor prognosis group (mRS > 2, *n* = 32) and good prognosis group (mRS ≤ 2, n = 84)	Poor prognosis group had higher NIHSS scores, IL-6 levels, and NSE levels.	IL-6 ≥ 6.805 pg/mL and NSE ≥ 7.445 ng/mL were independently associated with poor prognosis in AIS patients.
Wytrykowska et al., 2016 [38]	Case–control study	86 participants (50 patients with early ischemic stroke and 36 control subjects with no previous stroke)	Higher levels of IL-1β (13.01 ± 13.87 vs. 7.83 ± 2.11) and IL-6 (15.82 ± 16.64 vs. 6.64 ± 2.5) in ischemic stroke patients compared to controls.	Ischemic stroke is associated with elevated serum levels of IL-1β and IL-6.
Jiang et al., 2024 [39]	Observational study	305 patients admitted within 48 h of acute ischemic stroke onset	Each 1 pg/mL increase in IL-6 serum level was associated with an 8% increased risk of stroke recurrence (OR: 1.08; 95% CI: 1.04–1.11; *p* < 0.001).	IL-6 is a valuable predictive biomarker for stroke recurrence after acute ischemic stroke.
Meisinger et al., 2023 [40]	Cross-sectional study	364 patients with ischemic stroke from the Stroke Cohort Augsburg (SCHANA), classified by TOAST subtypes	Lower levels of interleukin-6 (IL-6) (β = −0.53; Padj = 0.036) and macrophage migration inhibitory factor (MIF) (β = −0.52; Padj = 0.043) in small vessel stroke compared to large vessel stroke.	Different immune dysregulation patterns were observed across ischemic stroke subtypes, providing insights into the pathophysiology of stroke.
MMP				
Cárcel-Márquez et al., 2021 [41]	Mendelian randomization study	MEGASTROKE cohort: 440,328 participants. GODs cohort: 1791 participants.	Lower MMP-1 and MMP-12 levels associated with reduced risk of large-artery atherosclerosis subtype. Higher MMP-8 levels linked to increased risk of lacunar (small-vessel) stroke.	Specific MMPs (MMP-1, MMP-8, and MMP-12) are differentially associated with stroke subtypes, suggesting distinct pathophysiological roles.
Wen et al., 2014 [42]	Meta-analysis of 11 studies	MMP-1 -1607 1G/2G: 589 cases and 494 controls. MMP-3 -1612 5A/6A: 1817 cases and 1731 controls. MMP-9 -1562C/T: 540 cases and 547 controls.	MMP-1 -1607 1G/2G is risk factor for ischemic stroke under the dominant model. MMP-3 -1612 5A/6A is risk factor for ischemic stroke. MMP-9 -1562C/T is not associated with ischemic stroke.	The meta-analysis highlights the complex interplay between genetic polymorphisms in the MMP family and the risk of ischemic stroke.
Huang et al., 2017 [43]	Case–control study	640 patients with ischemic stroke (IS) and 637 age- and gender-matched healthy controls	MMP-3 (−1171 5A/6A) polymorphism was significantly associated with overall ischemic stroke risk. MMP-1 (−1607 1G/2G) polymorphism showed no overall IS association but was linked to the small-artery occlusion subtype. MMP-3 5A/6A + 5A/5A genotypes were associated with the large-artery atherosclerosis subtype.	MMP-1 (−1607 1G/2G) and MMP-3 (−1171 5A/6A) polymorphisms are linked to specific subtypes of ischemic stroke, suggesting a genetic predisposition that varies across ischemic stroke subtypes.
Chehaibi et al., 2014 [44]	Case–control study	196 patients with ischemic stroke and 192 individuals without ischemic stroke	MMP-1-1607 1G/2G polymorphism have not significant interaction with Type 2 Diabetes on Ischemic Stroke risk; MMP-12 polymorphisms are associated with a higher risk of ischemic stroke in diabetic patients.	These findings suggest MMP-12 polymorphisms could serve as potential markers for cerebrovascular disorders in diabetic patients.

AIS, acute ischemic control; NVAF, non-valvular atrial fibrillation; ROC, receiving curve analysis; MBE, malignant brain edema.

## References

[1] (2023). The Rising Global Burden of Stroke. eClinicalMedicine.

[2] Pu L., Wang L., Zhang R., Zhao T., Jiang Y., Han L. (2023). Projected Global Trends in Ischemic Stroke Incidence, Deaths and Disability-Adjusted Life Years From 2020 to 2030. Stroke.

[3] GBD 2019 Stroke Collaborators (2021). Global, Regional, and National Burden of Stroke and Its Risk Factors, 1990–2019: A Systematic Analysis for the Global Burden of Disease Study 2019. Lancet Neurol..

[4] Capirossi C., Laiso A., Renieri L., Capasso F., Limbucci N. (2023). Epidemiology, Organization, Diagnosis and Treatment of Acute Ischemic Stroke. Eur. J. Radiol. Open.

[5] Banerjee C., Chimowitz M.I. (2017). Stroke Caused by Atherosclerosis of the Major Intracranial Arteries. Circ. Res..

[6] Madaudo C., Coppola G., Parlati A.L.M., Corrado E. (2024). Discovering Inflammation in Atherosclerosis: Insights from Pathogenic Pathways to Clinical Practice. Int. J. Mol. Sci..

[7] Miceli G., Basso M.G., Pintus C., Pennacchio A.R., Cocciola E., Cuffaro M., Profita M., Rizzo G., Tuttolomondo A. (2024). Molecular Pathways of Vulnerable Carotid Plaques at Risk of Ischemic Stroke: A Narrative Review. Int. J. Mol. Sci..

[8] Gui Y., Zheng H., Cao R.Y. (2022). Foam Cells in Atherosclerosis: Novel Insights Into Its Origins, Consequences, and Molecular Mechanisms. Front. Cardiovasc. Med..

[9] Neels J.G., Gollentz C., Chinetti G. (2023). Macrophage Death in Atherosclerosis: Potential Role in Calcification. Front. Immunol..

[10] Wang T., Palucci D., Law K., Yanagawa B., Yam J., Butany J. (2012). Atherosclerosis: Pathogenesis and Pathology. Diagn. Histopathol..

[11] Tate A.R., Rao G.H. (2024). Inflammation: Is It a Healer, Confounder, or a Promoter of Cardiometabolic Risks?. Biomolecules.

[12] Virchow R.L.K. (1860). Cellular Pathology.

[13] Ross R., Glomset J.A. (1976). The Pathogenesis of Atherosclerosis (Second of Two Parts). N. Engl. J. Med..

[14] Ross R. (1999). Atherosclerosis—An Inflammatory Disease. N. Engl. J. Med..

[15] Tanase D.M., Valasciuc E., Gosav E.M., Ouatu A., Buliga-Finis O.N., Floria M., Maranduca M.A., Serban I.L. (2023). Portrayal of NLRP3 Inflammasome in Atherosclerosis: Current Knowledge and Therapeutic Targets. Int. J. Mol. Sci..

[16] Libby P. (2012). Inflammation in Atherosclerosis. Arterioscler. Thromb. Vasc. Biol..

[17] Popa-Fotea N.-M., Ferdoschi C.-E., Micheu M.-M. (2023). Molecular and Cellular Mechanisms of Inflammation in Atherosclerosis. Front. Cardiovasc. Med..

[18] Minelli S., Minelli P., Montinari M.R. (2020). Reflections on Atherosclerosis: Lesson from the Past and Future Research Directions. J. Multidiscip. Healthc..

[19] Ketelhuth D.F.J., Lutgens E., Bäck M., Binder C.J., Van den Bossche J., Daniel C., Dumitriu I.E., Hoefer I., Libby P., O’Neill L. (2019). Immunometabolism and Atherosclerosis: Perspectives and Clinical Significance: A Position Paper from the Working Group on Atherosclerosis and Vascular Biology of the European Society of Cardiology. Cardiovasc. Res..

[20] Zhao T.X., Mallat Z. (2019). Targeting the Immune System in Atherosclerosis: JACC State-of-the-Art Review. J. Am. Coll. Cardiol..

[21] Saigusa R., Winkels H., Ley K. (2020). T Cell Subsets and Functions in Atherosclerosis. Nat. Rev. Cardiol..

[22] Živković L., Asare Y., Bernhagen J., Dichgans M., Georgakis M.K. (2022). Pharmacological Targeting of the CCL2/CCR2 Axis for Atheroprotection: A Meta-Analysis of Preclinical Studies. Arterioscler. Thromb. Vasc. Biol..

[23] Döring Y., Jansen Y., Cimen I., Aslani M., Gencer S., Peters L.J.F., Duchene J., Weber C., van der Vorst E.P.C. (2020). B-Cell-Specific CXCR4 Protects Against Atherosclerosis Development and Increases Plasma IgM Levels. Circ. Res..

[24] Cimen I., Natarelli L., Abedi Kichi Z., Henderson J.M., Farina F.M., Briem E., Aslani M., Megens R.T.A., Jansen Y., Mann-Fallenbuchel E. (2023). Targeting a Cell-Specific microRNA Repressor of CXCR4 Ameliorates Atherosclerosis in Mice. Sci. Transl. Med..

[25] Pradeu T., Cooper E.L. (2012). The Danger Theory: 20 Years Later. Front. Immunol..

[26] Gorey S., McCabe J., Kelly P. (2023). Inflammation—The New Treatment Target for Ischaemic Stroke Prevention. Front. Stroke.

[27] Ridker P.M., Everett B.M., Thuren T., MacFadyen J.G., Chang W.H., Ballantyne C., Fonseca F., Nicolau J., Koenig W., Anker S.D. (2017). Antiinflammatory Therapy with Canakinumab for Atherosclerotic Disease. N. Engl. J. Med..

[28] Wang X., Kou W., Kong W., Ma S., Xue Q., Zou Y., Song A. (2022). Magnetic Resonance Images, Pathological Features of Thrombus, and Expression of NLRP Inflammasome in Patients with Acute Ischemic Stroke. Contrast Media Mol. Imaging.

[29] Sun J., Xu J., Yang Q. (2022). Expression and Predictive Value of NLRP3 in Patients with Atrial Fibrillation and Stroke. Am. J. Transl. Res..

[30] Wang Y., Huang H., He W., Zhang S., Liu M., Wu S. (2021). Association between Serum NLRP3 and Malignant Brain Edema in Patients with Acute Ischemic Stroke. BMC Neurol..

[31] Duan R., Wang N., Shang Y., Li H., Liu Q., Li L., Zhao X. (2022). TNF-α (G-308A) Polymorphism, Circulating Levels of TNF-α and IGF-1: Risk Factors for Ischemic Stroke-An Updated Meta-Analysis. Front. Aging Neurosci..

[32] Loga-Andrijić N., Petrović N.T., Filipović-Danić S., Marjanović S., Mitrović V., Loga-Zec S. (2021). The Significance of Interleukin-6 and Tumor Necrosis Factor-Alpha Levels in Cognitive Impairment among First-Ever Acute Ischaemic Stroke Patients. Psychiatr. Danub..

[33] Wu J.-C., Zhang X., Wang J.-H., Liu Q.-W., Wang X.-Q., Wu Z.-Q., Wang J., Zhang C., Qun S. (2019). Gene Polymorphisms and Circulating Levels of the TNF-Alpha Are Associated with Ischemic Stroke: A Meta-Analysis Based on 19,873 Individuals. Int. Immunopharmacol..

[34] Georgakis M.K., Malik R., Björkbacka H., Pana T.A., Demissie S., Ayers C., Elhadad M.A., Fornage M., Beiser A.S., Benjamin E.J. (2019). Circulating Monocyte Chemoattractant Protein-1 and Risk of Stroke: Meta-Analysis of Population-Based Studies Involving 17,180 Individuals. Circ. Res..

[35] Cheng X., Lian Y.-J., Ma Y.-Q., Xie N.-C., Wu C.-J. (2017). Elevated Serum Levels of CXC Chemokine Ligand-12 Are Associated with Unfavorable Functional Outcome and Mortality at 6-Month Follow-up in Chinese Patients with Acute Ischemic Stroke. Mol. Neurobiol..

[36] Duan X.-X., Zhang G.-P., Wang X.-B., Yu H., Wu J.-L., Liu K.-Z., Wang L., Long X. (2015). The Diagnostic and Prognostic Value of Serum CXCL12 Levels in Patients with Ischemic Stroke. Neurol. Sci..

[37] Zhang M., Zhao H., Lu N., Zhang S. (2024). Predictive Value of Interleukin-6 Combined with Serum Neuron-Specific Enolase on the Prognosis of Acute Ischemic Stroke. Clin. Neurol. Neurosurg..

[38] Wytrykowska A., Prosba-Mackiewicz M., Nyka W.M. (2016). IL-1β, TNF-α, and IL-6 Levels in Gingival Fluid and Serum of Patients with Ischemic Stroke. J. Oral. Sci..

[39] Jiang Y., Fan T. (2024). IL-6 and Stroke Recurrence in Ischemic Stroke. Biomark. Med..

[40] Meisinger C., Freuer D., Schmitz T., Ertl M., Zickler P., Naumann M., Linseisen J. (2024). Inflammation Biomarkers in Acute Ischemic Stroke According to Different Etiologies. Eur. J. Neurol..

[41] Cárcel-Márquez J., Cullell N., Muiño E., Gallego-Fabrega C., Lledós M., Ibañez L., Krupinski J., Montaner J., Cruchaga C., Lee J.-M. (2021). Causal Effect of MMP-1 (Matrix Metalloproteinase-1), MMP-8, and MMP-12 Levels on Ischemic Stroke: A Mendelian Randomization Study. Stroke.

[42] Wen D., Du X., Nie S.-P., Dong J.-Z., Ma C.-S. (2014). Association between Matrix Metalloproteinase Family Gene Polymorphisms and Ischemic Stroke: A Meta-Analysis. Mol. Neurobiol..

[43] Huang X.-Y., Han L.-Y., Huang X.-D., Guan C.-H., Mao X.-L., Ye Z.-S. (2017). Association of Matrix Metalloproteinase-1 and Matrix Metalloproteinase-3 Gene Variants with Ischemic Stroke and Its Subtype. J. Stroke Cerebrovasc. Dis..

[44] Chehaibi K., Hrira M.Y., Nouira S., Maatouk F., Ben Hamda K., Slimane M.N. (2014). Matrix Metalloproteinase-1 and Matrix Metalloproteinase-12 Gene Polymorphisms and the Risk of Ischemic Stroke in a Tunisian Population. J. Neurol. Sci..

[45] Koliaki C., Dalamaga M., Liatis S. (2023). Update on the Obesity Epidemic: After the Sudden Rise, Is the Upward Trajectory Beginning to Flatten?. Curr. Obes. Rep..

[46] Gallo G., Desideri G., Savoia C. (2024). Update on Obesity and Cardiovascular Risk: From Pathophysiology to Clinical Management. Nutrients.

[47] Purnell J.Q., Feingold K.R., Anawalt B., Blackman M.R., Boyce A., Chrousos G., Corpas E., de Herder W.W., Dhatariya K., Dungan K., Hofland J. (2000). Definitions, Classification, and Epidemiology of Obesity. Endotext.

[48] National Institutes of Health (1998). Clinical Guidelines on the Identification, Evaluation, and Treatment of Overweight and Obesity in Adults—The Evidence Report. Obes. Res..

[49] Kolb H. (2022). Obese Visceral Fat Tissue Inflammation: From Protective to Detrimental?. BMC Med..

[50] Kolb H., Stumvoll M., Kramer W., Kempf K., Martin S. (2018). Insulin Translates Unfavourable Lifestyle into Obesity. BMC Med..

[51] Lemmer I.L., Willemsen N., Hilal N., Bartelt A. (2021). A Guide to Understanding Endoplasmic Reticulum Stress in Metabolic Disorders. Mol. Metab..

[52] Stenkula K.G., Erlanson-Albertsson C. (2018). Adipose Cell Size: Importance in Health and Disease. Am. J. Physiol. Regul. Integr. Comp. Physiol..

[53] Obradovic M., Sudar-Milovanovic E., Soskic S., Essack M., Arya S., Stewart A.J., Gojobori T., Isenovic E.R. (2021). Leptin and Obesity: Role and Clinical Implication. Front. Endocrinol..

[54] Kiernan K., MacIver N.J. (2020). The Role of the Adipokine Leptin in Immune Cell Function in Health and Disease. Front. Immunol..

[55] Trayhurn P. (2013). Hypoxia and Adipose Tissue Function and Dysfunction in Obesity. Physiol. Rev..

[56] Trim W.V., Walhin J.-P., Koumanov F., Bouloumié A., Lindsay M.A., Travers R.L., Turner J.E., Thompson D. (2022). The Impact of Long-Term Physical Inactivity on Adipose Tissue Immunometabolism. J. Clin. Endocrinol. Metab..

[57] Rausch M.E., Weisberg S., Vardhana P., Tortoriello D.V. (2008). Obesity in C57BL/6J Mice Is Characterized by Adipose Tissue Hypoxia and Cytotoxic T-Cell Infiltration. Int. J. Obes..

[58] Song A., Dai W., Jang M.J., Medrano L., Li Z., Zhao H., Shao M., Tan J., Li A., Ning T. (2020). Low- and High-Thermogenic Brown Adipocyte Subpopulations Coexist in Murine Adipose Tissue. J. Clin. Investig..

[59] Adachi Y., Ueda K., Nomura S., Ito K., Katoh M., Katagiri M., Yamada S., Hashimoto M., Zhai B., Numata G. (2022). Beiging of Perivascular Adipose Tissue Regulates Its Inflammation and Vascular Remodeling. Nat. Commun..

[60] Cheong L.Y., Xu A. (2021). Intercellular and Inter-Organ Crosstalk in Browning of White Adipose Tissue: Molecular Mechanism and Therapeutic Complications. J. Mol. Cell Biol..

[61] Huang Z., Xu A. (2021). Adipose Extracellular Vesicles in Intercellular and Inter-Organ Crosstalk in Metabolic Health and Diseases. Front. Immunol..

[62] Hill D.A., Lim H.-W., Kim Y.H., Ho W.Y., Foong Y.H., Nelson V.L., Nguyen H.C.B., Chegireddy K., Kim J., Habertheuer A. (2018). Distinct Macrophage Populations Direct Inflammatory versus Physiological Changes in Adipose Tissue. Proc. Natl. Acad. Sci. USA.

[63] Liang L., Chai Y., Chai F., Liu H., Ma N., Zhang H., Zhang S., Nong L., Li T., Zhang B. (2022). Expression of SASP, DNA Damage Response, and Cell Proliferation Factors in Early Gastric Neoplastic Lesions: Correlations and Clinical Significance. Pathol. Oncol. Res..

[64] Grosse L., Wagner N., Emelyanov A., Molina C., Lacas-Gervais S., Wagner K.-D., Bulavin D.V. (2020). Defined p16High Senescent Cell Types are Indispensable for Mouse Healthspan. Cell Metab..

[65] Hildreth A.D., Ma F., Wong Y.Y., Sun R., Pellegrini M., O’Sullivan T.E. (2021). Single-Cell Sequencing of Human White Adipose Tissue Identifies New Cell States in Health and Obesity. Nat. Immunol..

[66] American Diabetes Association Professional Practice Committee 9 (2024). Pharmacologic Approaches to Glycemic Treatment: Standards of Care in Diabetes-2024. Diabetes Care.

[67] Stefanovski D., Punjabi N.M., Boston R.C., Watanabe R.M. (2021). Insulin Action, Glucose Homeostasis and Free Fatty Acid Metabolism: Insights From a Novel Model. Front. Endocrinol..

[68] Maida C.D., Daidone M., Pacinella G., Norrito R.L., Pinto A., Tuttolomondo A. (2022). Diabetes and Ischemic Stroke: An Old and New Relationship an Overview of the Close Interaction between These Diseases. Int. J. Mol. Sci..

[69] Nițulescu I.M., Ciulei G., Cozma A., Procopciuc L.M., Orășan O.H. (2023). From Innate Immunity to Metabolic Disorder: A Review of the NLRP3 Inflammasome in Diabetes Mellitus. J. Clin. Med..

[70] Gu C., Pang B., Sun S., An C., Wu M., Wang N., Yuan Y., Liu G. (2023). Neutrophil Extracellular Traps Contributing to Atherosclerosis: From Pathophysiology to Clinical Implications. Exp. Biol. Med..

[71] Klopf J., Brostjan C., Eilenberg W., Neumayer C. (2021). Neutrophil Extracellular Traps and Their Implications in Cardiovascular and Inflammatory Disease. Int. J. Mol. Sci..

[72] Iqbal A.M., Jamal S.F. (2024). Essential Hypertension. StatPearls.

[73] Yusuf S., Joseph P., Rangarajan S., Islam S., Mente A., Hystad P., Brauer M., Kutty V.R., Gupta R., Wielgosz A. (2020). Modifiable Risk Factors, Cardiovascular Disease, and Mortality in 155,722 Individuals from 21 High-Income, Middle-Income, and Low-Income Countries (PURE): A Prospective Cohort Study. Lancet.

[74] O’Donnell M.J., Xavier D., Liu L., Zhang H., Chin S.L., Rao-Melacini P., Rangarajan S., Islam S., Pais P., McQueen M.J. (2010). Risk Factors for Ischaemic and Intracerebral Haemorrhagic Stroke in 22 Countries (the INTERSTROKE Study): A Case-Control Study. Lancet.

[75] Webb A.J.S., Werring D.J. (2022). New Insights Into Cerebrovascular Pathophysiology and Hypertension. Stroke.

[76] Virani S.S., Alonso A., Aparicio H.J., Benjamin E.J., Bittencourt M.S., Callaway C.W., Carson A.P., Chamberlain A.M., Cheng S., Delling F.N. (2021). Heart Disease and Stroke Statistics-2021 Update: A Report From the American Heart Association. Circulation.

[77] Di Chiara T., Del Cuore A., Daidone M., Scaglione S., Norrito R.L., Puleo M.G., Scaglione R., Pinto A., Tuttolomondo A. (2022). Pathogenetic Mechanisms of Hypertension-Brain-Induced Complications: Focus on Molecular Mediators. Int. J. Mol. Sci..

[78] Chatterjee S. (2018). Endothelial Mechanotransduction, Redox Signaling and the Regulation of Vascular Inflammatory Pathways. Front. Physiol..

[79] Gallo G., Volpe M., Savoia C. (2021). Endothelial Dysfunction in Hypertension: Current Concepts and Clinical Implications. Front. Med..

[80] Griendling K.K., Camargo L.L., Rios F.J., Alves-Lopes R., Montezano A.C., Touyz R.M. (2021). Oxidative Stress and Hypertension. Circ. Res..

[81] De Silva T.M., Modrick M.L., Grobe J.L., Faraci F.M. (2021). Activation of the Central Renin-Angiotensin System Causes Local Cerebrovascular Dysfunction. Stroke.

[82] Guo Q., Jin Y., Chen X., Ye X., Shen X., Lin M., Zeng C., Zhou T., Zhang J. (2024). NF-κB in Biology and Targeted Therapy: New Insights and Translational Implications. Signal Transduct. Target. Ther..

[83] Amponsah-Offeh M., Diaba-Nuhoho P., Speier S., Morawietz H. (2023). Oxidative Stress, Antioxidants and Hypertension. Antioxidants.

[84] Salminen A., Kaarniranta K., Kauppinen A. (2013). Crosstalk between Oxidative Stress and SIRT1: Impact on the Aging Process. Int. J. Mol. Sci..

[85] Rizzoni D., Agabiti-Rosei C., De Ciuceis C. (2023). State of the Art Review: Vascular Remodeling in Hypertension. Am. J. Hypertens..

[86] Singh C.K., Chhabra G., Ndiaye M.A., Garcia-Peterson L.M., Mack N.J., Ahmad N. (2018). The Role of Sirtuins in Antioxidant and Redox Signaling. Antioxid. Redox Signal..

[87] Kim H.-L. (2023). Arterial Stiffness and Hypertension. Clin. Hypertens..

[88] Katsi V., Marketou M., Maragkoudakis S., Didagelos M., Charalambous G., Parthenakis F., Tsioufis C., Tousoulis D. (2020). Blood-Brain Barrier Dysfunction: The Undervalued Frontier of Hypertension. J. Hum. Hypertens..

[89] Du Z., Qin Y. (2023). Dyslipidemia and Cardiovascular Disease: Current Knowledge, Existing Challenges, and New Opportunities for Management Strategies. J. Clin. Med..

[90] Mohamed-Yassin M.-S., Baharudin N., Abdul-Razak S., Ramli A.S., Lai N.M. (2021). Global Prevalence of Dyslipidaemia in Adult Populations: A Systematic Review Protocol. BMJ Open.

[91] de Oliveira J.V.B., Lima R.P.A., Pordeus Luna R.C., da Silva Diniz A., de Almeida A.T.C., de Oliveira N.F.P., Gonçalves M.d.C.R., de Lima R.T., de Lima Ferreira F.E.L., Diniz S.C.P.d.O.R. (2020). The Direct Correlation between Oxidative Stress and LDL-C Levels in Adults Is Maintained by the Friedewald and Martin Equations, but the Methylation Levels in the MTHFR and ADRB3 Genes Differ. PLoS ONE.

[92] Njeim R., Alkhansa S., Fornoni A. (2023). Unraveling the Crosstalk between Lipids and NADPH Oxidases in Diabetic Kidney Disease. Pharmaceutics.

[93] Faria-Neto J.R., Chyu K.-Y., Li X., Dimayuga P.C., Ferreira C., Yano J., Cercek B., Shah P.K. (2006). Passive Immunization with Monoclonal IgM Antibodies against Phosphorylcholine Reduces Accelerated Vein Graft Atherosclerosis in Apolipoprotein E-Null Mice. Atherosclerosis.

[94] Su J., Georgiades A., Wu R., Thulin T., de Faire U., Frostegård J. (2006). Antibodies of IgM Subclass to Phosphorylcholine and Oxidized LDL Are Protective Factors for Atherosclerosis in Patients with Hypertension. Atherosclerosis.

[95] Higashi Y. (2023). Endothelial Function in Dyslipidemia: Roles of LDL-Cholesterol, HDL-Cholesterol and Triglycerides. Cells.

[96] Gordon T., Castelli W.P., Hjortland M.C., Kannel W.B., Dawber T.R. (1977). High Density Lipoprotein as a Protective Factor against Coronary Heart Disease. The Framingham Study. Am. J. Med..

[97] Melin E.O., Thulesius H.O., Hillman M., Svensson R., Landin-Olsson M., Thunander M. (2019). Lower HDL-Cholesterol, a Known Marker of Cardiovascular Risk, Was Associated with Depression in Type 1 Diabetes: A Cross Sectional Study. Lipids Health Dis..

[98] Christensen A.A., Gannon M. (2019). The Beta Cell in Type 2 Diabetes. Curr. Diab Rep..

[99] Yamamoto W.R., Bone R.N., Sohn P., Syed F., Reissaus C.A., Mosley A.L., Wijeratne A.B., True J.D., Tong X., Kono T. (2019). Endoplasmic Reticulum Stress Alters Ryanodine Receptor Function in the Murine Pancreatic β Cell. J. Biol. Chem..

[100] Halban P.A., Polonsky K.S., Bowden D.W., Hawkins M.A., Ling C., Mather K.J., Powers A.C., Rhodes C.J., Sussel L., Weir G.C. (2014). β-Cell Failure in Type 2 Diabetes: Postulated Mechanisms and Prospects for Prevention and Treatment. J. Clin. Endocrinol. Metab..

[101] Darenskaya M.A., Kolesnikova L.I., Kolesnikov S.I. (2021). Oxidative Stress: Pathogenetic Role in Diabetes Mellitus and Its Complications and Therapeutic Approaches to Correction. Bull. Exp. Biol. Med..

[102] Guthrie R.A., Guthrie D.W. (2004). Pathophysiology of Diabetes Mellitus. Crit. Care Nurs. Q..

[103] Libby P. (2024). Inflammation and the Pathogenesis of Atherosclerosis. Vasc. Pharmacol..

[104] Kong P., Cui Z.-Y., Huang X.-F., Zhang D.-D., Guo R.-J., Han M. (2022). Inflammation and Atherosclerosis: Signaling Pathways and Therapeutic Intervention. Signal Transduct. Target. Ther..

[105] Vengrenyuk Y., Nishi H., Long X., Ouimet M., Savji N., Martinez F.O., Cassella C.P., Moore K.J., Ramsey S.A., Miano J.M. (2015). Cholesterol Loading Reprograms the microRNA-143/145-Myocardin Axis to Convert Aortic Smooth Muscle Cells to a Dysfunctional Macrophage-like Phenotype. Arterioscler. Thromb. Vasc. Biol..

[106] De Vriese A.S., Verbeuren T.J., Van de Voorde J., Lameire N.H., Vanhoutte P.M. (2000). Endothelial Dysfunction in Diabetes—De Vriese—2000—British Journal of Pharmacology—Wiley Online Library. Br. J. Pharmacol..

[107] Milstien S., Katusic Z. (1999). Oxidation of Tetrahydrobiopterin by Peroxynitrite: Implications for Vascular Endothelial Function. Biochem. Biophys. Res. Commun..

[108] Inoguchi T., Li P., Umeda F., Yu H.Y., Kakimoto M., Imamura M., Aoki T., Etoh T., Hashimoto T., Naruse M. (2000). High Glucose Level and Free Fatty Acid Stimulate Reactive Oxygen Species Production through Protein Kinase C—Dependent Activation of NAD(P)H Oxidase in Cultured Vascular Cells. Diabetes.

[109] Katakami N. (2018). Mechanism of Development of Atherosclerosis and Cardiovascular Disease in Diabetes Mellitus. J. Atheroscler. Thromb..

[110] Kaur R., Kaur M., Singh J. (2018). Endothelial Dysfunction and Platelet Hyperactivity in Type 2 Diabetes Mellitus: Molecular Insights and Therapeutic Strategies. Cardiovasc. Diabetol..

[111] Carr M.E. (2001). Diabetes Mellitus: A Hypercoagulable State. J. Diabetes Complicat..

[112] Li X., Weber N.C., Cohn D.M., Hollmann M.W., DeVries J.H., Hermanides J., Preckel B. (2021). Effects of Hyperglycemia and Diabetes Mellitus on Coagulation and Hemostasis. J. Clin. Med..

[113] Barone F.C., Feuerstein G.Z. (1999). Inflammatory mediators and stroke: New opportunities for novel therapeutics. J. Cereb. Blood Flow Metabolism..

[114] Chamorro A., Hallenbeck J. (2006). The harms and benefits of inflammatory and immune responses in vascular disease. Stroke.

[115] Davies C.A., Loddick S.A., Stroemer R.P., Hunt J., Rothwell N.J. (1998). An integrated analysis of the progression of cell responses induced by permanent focal middle cerebral artery occlusion in the rat. Exp. Neurol..

[116] Tuttolomondo A., Pedone C., Pinto A., Di Raimondo D., Fernandez P., Di Sciacca R., Licata G. (2008). Gruppo Italiano di Farmacoepidemiologia dell’Anziano (GIFA) researchers. Predictors of outcome in acute ischemic cerebrovascular syndromes: The GIFA study. Int. J. Cardiol..

[117] Davì G., Tuttolomondo A., Santilli F., Basili S., Ferrante E., Di Raimondo D., Pinto A., Licata G. (2009). CD40 ligand and MCP-1 as predictors of cardiovascular events in diabetic patients with stroke. J. Atheroscler. Thromb..

[118] Della Corte V., Tuttolomondo A., Pecoraro R., Di Raimondo D., Vassallo V., Pinto A. (2016). Inflammation, Endothelial Dysfunction and Arterial Stiffness as Therapeutic Targets in Cardiovascular Medicine. Curr. Pharm. Des..

[119] Tuttolomondo A., Petta S., Casuccio A., Maida C., Corte V.D., Daidone M., Di Raimondo D., Pecoraro R., Fonte R., Cirrincione A. (2018). Reactive hyperemia index (RHI) and cognitive performance indexes are associated with histologic markers of liver disease in subjects with non-alcoholic fatty liver disease (NAFLD): A case control study. Cardiovasc. Diabetol..

[120] Suzuki H., Abe K., Tojo S., Morooka S., Kimura K., Mizugaki M., Itoyama Y. (1997). Postischemic expression of P-selectin immunore-activity in rat brain. Neurosci. Lett..

[121] Hallenbeck J.M. (1996). Significance of the inflammatory response in brain ischemia. Acta Neurochir. Suppl..

[122] Ma W.Y., Wu Q.L., Wang S.S., Wang H.Y., Ye J.R., Sun H.S., Feng Z.P., He W.B., Chu S.F., Zhang Z. (2023). A breakdown of metabolic reprogramming in microglia induced by CKLF1 exacerbates immune tolerance in ischemic stroke. J. Neuroinflammation.

[123] Soto-Cámara R., González-Santos J., González-Berna J., Trejo-Gabriel-Galán J.M. (2020). Factors Associated with a Rapid Call for Assistance for Patients with Ischemic Stroke. Emergencias.

[124] Soto-Cámara R., González-Bernal J.J., González-Santos J., Aguilar-Parra J.M., Trigueros R., López-Liria R. (2020). Age-Related Risk Factors at the First Stroke Event. J. Clin. Med..

[125] Jia Q., Zhao X., Wang C., Wang Y., Yan Y., Li H., Zhong L., Liu L., Zheng H., Zhou Y. (2011). Diabetes and Poor Outcomes within 6 Months after Acute Ischemic Stroke: The China National Stroke Registry. Stroke.

[126] Hankey G.J., Anderson N.E., Ting R.-D., Veillard A.-S., Romo M., Wosik M., Sullivan D.R., O’Connell R.L., Hunt D., Keech A.C. (2013). Rates and Predictors of Risk of Stroke and Its Subtypes in Diabetes: A Prospective Observational Study. J. Neurol. Neurosurg. Psychiatry.

[127] Lindsberg P.J., Roine R.O. (2004). Hyperglycemia in Acute Stroke. Stroke.

[128] Reinke C., Buchmann N., Fink A., Tegeler C., Demuth I., Doblhammer G. (2022). Diabetes Duration and the Risk of Dementia: A Cohort Study Based on German Health Claims Data. Ageing.

[129] Nazir F.S., Alem M., Small M., Connell J.M.C., Lees K.R., Walters M.R., Cleland S.J. (2006). Blunted Response to Systemic Nitric Oxide Synthase Inhibition in the Cerebral Circulation of Patients with Type 2 Diabetes. Diabet. Med..

[130] Novak V., Zhao P., Manor B., Sejdic E., Alsop D., Abduljalil A., Roberson P.K., Munshi M., Novak P. (2011). Adhesion Molecules, Altered Vasoreactivity, and Brain Atrophy in Type 2 Diabetes. Diabetes Care.

[131] Wang D., Pan Y., Li H., Yan H., Meng X., Lin J., Wang H., Matsushita K., Shlipak M.G., Zhou Y. (2022). The Association between Baseline and 3-Month Albuminuria and 1-Year Prognosis of Ischemic Stroke. Cerebrovasc. Dis..

[132] Kalaria R.N., Akinyemi R., Ihara M. (2016). Stroke Injury, Cognitive Impairment and Vascular Dementia. Biochim. Biophys. Acta.

[133] Tang S.-C., Yang K.-C., Hu C.-J., Chiou H.-Y., Wu C.C., Jeng J.-S. (2017). Elevated Plasma Level of Soluble Form of RAGE in Ischemic Stroke Patients with Dementia. Neuromolecular Med..

[134] van Sloten T.T., Sedaghat S., Carnethon M.R., Launer L.J., Stehouwer C.D.A. (2020). Cerebral Microvascular Complications of Type 2 Diabetes: Stroke, Cognitive Dysfunction, and Depression. Lancet Diabetes Endocrinol..

[135] Ouk M., Wu C.-Y., Colby-Milley J., Fang J., Zhou L., Shah B.R., Herrmann N., Lanctôt K.L., Linkewich E., Law M. (2020). Depression and Diabetes Mellitus Multimorbidity Is Associated with Loss of Independence and Dementia Poststroke. Stroke.

[136] Liu W., Wong A., Au L., Yang J., Wang Z., Leung E.Y.L., Chen S., Ho C.L., Mok V.C.T. (2015). Influence of Amyloid-β on Cognitive Decline After Stroke/Transient Ischemic Attack: Three-Year Longitudinal Study. Stroke.

